# The Role of MicroRNA in the Behaviour of Periodontal Ligament Stem Cells and Stem Cells from the Apical Papilla: A Systematic Review

**DOI:** 10.1007/s12015-026-11088-7

**Published:** 2026-03-12

**Authors:** José Luis Sanz, Leopoldo Forner, Sergio López-García, Francisco Javier Rodríguez-Lozano, João Miguel Santos, Sofía Folguera, Concha López-Ginés, Daniel Monleón

**Affiliations:** 1 Department of Stomatology, Faculty of Medicine and Dentistry, Universitat de València, 46010 Valencia, Spain; 2 Biomedical Research Institute of Murcia (IMIB-Pascual Parrilla), Murcia, Spain; 3 Department of Dermatology, Stomatology, Radiology and Physical Medicine, Morales Meseguer Hospital, Faculty of Medicine, University of Murcia, 30100 Murcia, Spain; 4 Institute of Endodontics, Faculty of Medicine, University of Coimbra, 3000-075 Coimbra, Portugal; 5 Department of Pathology, Faculty of Medicine and Dentistry, Universitat de València, 46010 Valencia, Spain

**Keywords:** MicroRNA, Periodontal ligament stem cells, Stem cells from the apical papilla, Dental stem cells, Signaling pathway, Differentiation

## Abstract

**Aim:**

This systematic review aimed to synthesize current evidence on the regulatory role of microRNAs (miRNAs) in the viability, proliferation, osteo-odontogenic differentiation potential and/or inflammation of periodontal ligament stem cells (PDLSCs) and stem cells from the apical papilla (SCAPs), with a focus on their potential in periodontal and endodontic regeneration.

**Methodology:**

A comprehensive search across Medline, Scopus, Embase, Web of Science, and SciELO databases up to December 2025 identified original in vitro studies assessing miRNA overexpression or knockdown in PDLSCs or SCAPs. 39 studies met the eligibility criteria and underwent structured data extraction, qualitative synthesis and quality appraisal using a tailored risk-of-bias tool (miRoB-DSC).

**Results:**

The findings demonstrate that specific miRNAs act as key regulators of PDLSCs and SCAPs viability, proliferation, osteo-odontogenic differentiation, and inflammatory responses. Comparisons with previous reviews on DPSCs and PDLSCs suggest both shared and niche-specific regulatory networks. Various signaling pathways have been majorly implicated with miRNA regulation, including RUNX2, Smad/TGFβ, NOTCH, NF-κB, Wnt/β-catenin and MAPK. The majority of the assessed studies fulfilled more than 80% of the applicable items from the miRoB-DSC tool.

**Conclusion:**

Collectively, these results highlight miRNAs as central modulators of PDLSC and SCAP biology, with potential applications as therapeutic targets or biomarkers in regenerative dentistry. However, heterogeneity in experimental designs, limited evaluation under disease-relevant conditions, and the reliance on in vitro models highlight the need for standardized protocols and in vivo validation before clinical translation.

**Supplementary Information:**

The online version contains supplementary material available at 10.1007/s12015-026-11088-7.

## Introduction

Since their first isolation and culture from the periodontal ligament of extracted third molars [1], periodontal ligament stem cells (PDLSCs) have attracted considerable interest as candidates for stem cell-based therapies and biologically driven regenerative procedures. PDLSCs are a subset of dental stem cells (DSCs) that exhibit a mesenchymal stem cell (MSC) phenotype, characterized by the expression of MSC-associated surface markers (CD10, CD13, CD29, CD44, CD59, CD73, CD90, CD105, CD146, CD166) and the absence of hematopoietic (CD14, CD34, CD45, CD79a) and endothelial markers (CD31), as well as co-stimulatory molecules involved in immune activation (HLA-DR, CD40, CD54, CD80, CD86) [2–7]. In addition to their well-defined immunophenotype, PDLSCs demonstrate osteogenic, adipogenic, and chondrogenic differentiation in vitro [8–10], underscoring their multipotency and potential for tissue regeneration.

Beyond their multipotent differentiation ability, PDLSCs are increasingly positioned as key “seed cells” for periodontal tissue engineering, with recent reviews synthesizing preclinical and early clinical evidence that they contribute to coordinated regeneration of the cementum-periodontal ligament-bone complex and mediate the inflamed periodontal microenvironment via immunoregulatory and paracrine actions [11–13]. At the translational level, recent studies report that dental MSC-based approaches, including PDLSC strategies, are biologically plausible and generally safe, but current human evidence remains heterogeneous and underpowered, highlighting the need for larger, standardized trials before routine clinical use [14, 15]. On the other hand, cell-free strategies i.e. those involving the use of products derived from stem cells, are gaining popularity. Reviews on PDLSC-derived extracellular vesicles/exosomes emphasize their capacity to enhance osteogenesis/angiogenesis and influence inflammation, especially when integrated with advanced biomaterial scaffolds, offering a potentially easier alternative to cell transplantation [16, 17]. Collectively, the current literature frames PDLSCs as a versatile option for biologically based periodontal regeneration, with converging progress in delivery formats (e.g., cell sheets/pellets, electrospun/3D-printed scaffolds) and in mechanistic understanding of paracrine and miRNA-mediated effects [18–20].

In parallel, the apical papilla, a specialized soft tissue structure located around the apex of immature permanent teeth which plays a crucial role in root development and dentinogenesis, hosts another population of multipotent postnatal stem cells which also exhibit a mesenchymal nature: stem cells from the apical papilla (SCAPs) [21, 22]. SCAPs possess the ability to differentiate into odontogenic, osteogenic, adipogenic, and neurogenic lineages [23], but their most distinctive feature lies in their survival in dental pulp inflammation, suggesting that the apical papilla provides a relatively stable environment for SCAPs to maintain their vitality and stemness [24].

Thus, translational research aims to explore their use in pulp-dentin complex regeneration [25]. Specifically, their differentiation potential is exploited in regenerative endodontic treatment (RET), a novel biologically-based approach which aims for the continued root development of necrotic immature permanent teeth as an alternative to traditional apexification procedures [26, 27]. The biological basis for RET involves SCAP homing via the formation of a blood clot from the periapical tissues inside a previously disinfected root canal. Together with a biomaterial used as a coronal barrier to protect the blood clot and provide a bioactive medium, SCAPs may differentiate into odontoblast-like cells and foster mineralized tissue neo-formation [28, 29].

In addition, recent reviews highlight that DSCs, including SCAPs, exert paracrine and immunomodulatory effects through the release of extracellular vesicles and microRNAs, suggesting that their therapeutic impact may extend beyond direct differentiation to include regulation of angiogenesis, neurogenesis, and osteogenesis in the periapical environment [30, 31]. Together with evidence from PDLSCs, these findings underline that the regenerative activity of dental stem cells may depend as much on their secretome, particularly extracellular vesicle-encapsulated microRNAs, as on their direct differentiation capacity.

MicroRNAs (miRNAs) are short, non-coding RNAs of approximately 20–30 nucleotides (most commonly ~ 22 nucleotides) that regulate gene expression post-transcriptionally by binding to complementary sequences on target mRNAs, resulting in translational repression or degradation [32, 33]. They function as critical molecular regulators of stem cell biology, influencing proliferation, differentiation, apoptosis, and immunomodulation. Importantly, miRNAs operate not only within cells but can also be secreted via extracellular vesicles such as exosomes, thereby mediating intercellular communication in tissue homeostasis and regeneration [20, 34].

In the context of DSCs, miRNAs have been shown to regulate multiple signalling pathways, including Wnt/β-catenin, BMP/Smad, MAPK, and NF-κB, that are central to osteogenic, odontogenic, and angiogenic differentiation [35, 36]. Exosomal miRNAs released by PDLSCs and SCAPs are now recognized as key paracrine effectors that can shape the inflammatory microenvironment, promote angiogenesis, and enhance mineralized tissue formation, underscoring their therapeutic potential in periodontal and endodontic regeneration [17, 31, 37]. Furthermore, pathological conditions such as inflammation, diabetes, and mechanical stress alter the miRNA expression profiles of DSCs, which may impair or, conversely, enhance their regenerative performance depending on the context [38, 39].

Despite these advances, the available evidence remains fragmented across different stem cell types, microenvironments, and outcome measures. While previous reviews have focused primarily on dental pulp stem cells (DPSCs), there is still a lack of systematic synthesis addressing the broad role of miRNAs in the behaviour of other DSCs, such as PDLSCs and SCAPs. The present work therefore aims to systematically evaluate and integrate current evidence on the regulatory role of miRNAs in the viability, proliferation, osteo-odontogenic differentiation potential and/or inflammation of these two dental stem cell populations, focusing on their contribution to dental tissue regeneration. Specifically, this systematic review aims to identify specific miRNAs which have been shown to regulate PLDSC and/or SCAP behaviour and the implicated signalling pathways, whenever possible. Additionally, comparisons with previous evidence on the influence of miRNAs with other DSC subtypes will be made to elucidate the shared and/or niche-specific nature of the identified regulatory networks.

## Materials and Methods

This systematic review was performed under the framework of the PRISMA 2020 (Preferred Reporting Items for Systematic Reviews and Meta-Analyses) statement guidelines [40]. The protocol for the present work was registered in Open Science Framework (OSF) Registries and can be accessed with the following registration 10.17605/OSF.IO/AQ2N5 .The following protocol for the study search and selection, data extraction, and quality assessment was performed parallelly by two independent researchers. In the event of any inconsistency, a third investigator was consulted.

### Eligibility Criteria

Original studies analysing the role of one or more miRNAs on PDLSC and/or SCAP behaviour were considered for inclusion. The assessment of the ‘role’ of the miRNA was defined as the effect of their knockdown or overexpression on the tested cells. The assessment of the cellular ‘behaviour’ was defined as any measurement of cell viability, proliferation, differentiation potential (i.e. osteo/cemento/odontogenic) and/or inflammation.

The PICOS model [41] was used to develop the search question and study eligibility, as follows: population (P): PDLSCs and/or SCAPs; intervention (I): knockdown or overexpression of one or more miRNA; comparison/control (C): non-modified PDLSCs and/or SCAPs; outcome (O): cell viability, proliferation, differentiation, and inflammation; study design (S): in vitro.

### Search Strategy and Terminology

An advanced search string was designed and applied in five electronic databases: Medline, Scopus, Embase, Web of Science, and SciELO. The search was last updated in the 7th of February 2026, with results limited to studies published up to and including 2025, and without language restrictions. The search strategy included the following terms: ‘periodontal ligament stem cells’, ‘PDLSC, ‘stem cells from the apical papilla, ‘SCAP, ‘microRNA, and ‘miRNA’; annexed using the Boolean operators ‘OR’ and ‘AND’. The keyword selection was based on previous studies in the field and their most cited descriptors. In addition, the references from included studies were manually screened after the selection process to check for additional potentially eligible works. The search strategy and the findings for both the independent and combined search fields are illustrated in Table 1.

**Table 1:** Search strategy and findings per database

Database	Search strategy	Findings
Medline	#1 “periodontal ligament stem cells” or PDLSC	1,755
	#2 “stem cells from the apical papilla” or SCAP	1,517
	#3 microRNA OR miRNA	207,100
	**(#1 OR #2) AND #3**	**198**
Scopus	#1 TITLE-ABS-KEY (“periodontal ligament stem cells” or PDLSC)	2,194
	#2 TITLE-ABS-KEY (“stem cells from the apical papilla” or SCAP)	2,103
	#3 TITLE-ABS-KEY (microRNA OR miRNA)	254,774
	**(#1 OR #2) AND #3**	**246**
Embase	#1 “periodontal ligament stem cells” or PDLSC	2,010
	#2 “stem cells from the apical papilla” or SCAP	2,004
	#3 microRNA OR miRNA	295,5853
	**(#1 OR #2) AND #3**	**247**
Web of Science Core Collection	#1 TS = (“periodontal ligament stem cells” or PDLSC)	1,601
	#2 TS = (“stem cells from the apical papilla” or SCAP)	1,643
	#3 TS = (microRNA OR miRNA)	169,357
	**(#1 OR #2) AND #3**	**127**
SciELO	#1 “periodontal ligament stem cells” or PDLSC	29
	#2 “stem cells from the apical papilla” or SCAP	22
	#3 microRNA OR miRNA	1083
	**(#1 OR #2) AND #3**	**0**

### Study Screening and Selection Process

The retrieved records were exported from each database into a reference manager software (Mendeley 1.19.8; Elsevier, AMS, Netherlands), and duplicate records were manually discarded using the “check for duplicates” tool. Then, an initial screening of the titles and abstracts of the resulting records was performed using the previously established inclusion criteria. Lastly, a second screening of the full text of the articles which met the criteria was performed to confirm their eligibility.

### Data Extraction

Authors and years of publication were recorded as bibliometric parameters. Regarding the methodology of the included studies, the following variables were registered: tested DSC, microRNA(s) assessed (and their knockdown or overexpression), and in vitro assays performed to measure cellular behaviour. Lastly, the following outcome variables were registered: the interaction of microRNA(s) with their target marker (upregulation or downregulation), the associated pathway(s) in the interaction, their effect in PDLSC or SCAP behaviour, and the significant in vitro effects exhibited.

### Quality Assessment

No specific tool or reporting guidelines were found to assess the quality of the subtype of in vitro cellular studies included in this review. Consequently, an adapted checklist was developed based on in vitro-specific items from general tools in the field like the Toxicological data Reliability Assessment Tool (ToxRTool) [42], or the Quality Assessment Tool For In Vitro Studies (QUIN Tool) [43]. The acronym miRoB-DSC (MicroRNA Risk of Bias tool for Dental Stem Cell studies) was established for the tool. In brief, the following domains were assessed: 1) Cell Source and Characterization, 2) Experimental Design, 3) Outcome Assessment, 4) Data Reporting and Analysis, 5) Risk of Bias and Reproducibility. A total of 17 items were included among the different domains (Table 2). Studies were assessed for fullfillment or non-fulfillment of each of the items and a percentage of item fulfillment was calculated as a means of quality assessment i.e., the higher the percentage, the greater the transparency and reproducibility of the study. The explanation and elaboration of each Domain and Item, along with a template of the quality assessment tool checklist are presented as supplementary material.

**Table 2:** miRoB-DSC tool domains and items

Item n	Item question
**Domain 1. Cell Source and Characterization**	
1	Was the origin of the cells clearly described (tooth type, donor age/health, tissue source)?
2	Were cell identity and stemness markers verified (e.g., MSC markers, SCAP-specific CD24)?
3	Were passage number and culture conditions reported?
**Domain 2. Experimental Design**	
4	Were appropriate controls included (e.g., negative/positive controls)?
5	Were interventions (miRNA overexpression/inhibition) adequately described and validated?
6	Were sufficient biological replicates used (≥ 3 donors or independent experiments)?
7	Were treatment groups randomly assigned?
8	Were culture conditions standardized across groups?
**Domain 3. Outcome Assessment**	
9	Were relevant and validated outcome measures used (e.g., qPCR, Western blot, ALP, ARS staining, mineralization assays)?
10	Were outcome assessors blinded or were objective, automated methods applied?
11	Was normalization to housekeeping genes/proteins appropriately performed?
**Domain 4. Data Reporting and Analysis**	
12	Were statistical methods clearly described and appropriate for the design?
13	Were effect sizes and variability measures (e.g., SD/SEM) reported?
14	Was selective outcome reporting avoided (all planned outcomes reported)?
**Domain 5. Risk of Bias and Reproducibility**	
15	Were conflicts of interest declared, and funding sources disclosed?
16	Were raw data or supplementary datasets made available?
17	Were potential sources of bias and limitations discussed?

## Results

### Search and Study Selection Results

**Fig. 1:**
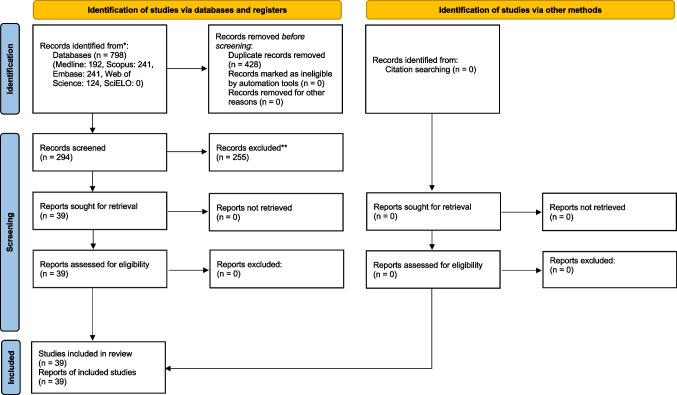
Study selection process. Based on the PRISMA 2020 [40] flow diagram

Figure 1 presents a flow diagram to illustrate the results from the database search ad study selection process. The electronic database searches identified a total of 818 records: (Medline: 198, Scopus: 246, Embase: 247, Web of Science: 127, SciELO: 0). No additional records were considered eligible upon the manual search of the references of the included studies. A total of 431 duplicate records were discarded. From the resulting 387 records, 347 were excluded after the initial screening of their titles and abstracts. All 40 of the resulting studies were considered as eligible for qualitative synthesis after full-text screening [44–83].

### Qualitative Synthesis

#### Influence of MiRNA on Cell Viability and Proliferation

The extracted data from the selected studies with regards to the role of miRNA in the regulation of PDLSC viability and proliferation are shown in Table 3. Only one study assessed the influence of miRNA on SCAP viability and proliferation. The overexpression of miR-142-3p, miR-375, miR-466, and miR‐146a, and the knockdown of miR-375 resulted in a reduced cell viability and/or proliferation of PDLSCs. Contrarily, an increased PDLSC viability and/or proliferation was observed with the overexpression of miR‑589‑3p and miR-375, and the knockdown of miR-671-5p, miR-142-3p, miR-152-3p. Regarding SCAPs, only one study observed that the overexpression of miR-141-3p resulted in a reduced cell viability and/or proliferation [51].

**Table 3:** Role of miRNA in the regulation of PDLSC viability and proliferation

Effect	MicroRNA	Target (interaction)	Significant in vitro effects (assay)	Reference
Viability and proliferation inhibition	miR-142-3p overexpression	SGK1 (-)	Reduced cell viability (CCK-8)	Sun et al. 2023 [77]
	miR-146a overexpression	-	Reduced cell viability (CCK-8)	Wang et al. 2021 [63]
	miR-375 knockdown	TOB2 (+)	Increased cell proliferation (MTT)	Wang et al. 2020 [60]
	miR-4661 overexpression	-	Reduced cell proliferation (CCK-8)	Liu et al. 2020 [57]
	miR‐146a overexpression	-	Reduced cell proliferation (CCK-8)	Zhao et al. 2019 [53]
Viability and proliferation promotion	miR-671-5p knockdown	DUSP8 (+)	Increased cell viability (CCK-8)	Wang et al. 2024 [78]
	miR-142-3p knockdown	SGK1 (+)	Increased cell viability (CCK-8)	Sun et al. 2023 [77]
	miR‑589‑3p overexpression	ATF1 (-)	Increased cell viability (CCK-8)	Shi et al. 2022 [74]
	miR-152-3p knockdown	ITGA5 (+)	Increased cell viability (CCK-8)	Wu & Ma 2020 [59]
	miR-375 overexpression	TOB2 (-)	Increased cell proliferation (MTT)	Wang et al. 2020 [60]

(+): miRNA increases target activity (upregulation/enhancement); (-): miRNA decreases target activity (downregulation/repression).

#### Influence of MiRNA on Cell Osteo-Odontogenic Differentiation

The extracted data from the selected studies with regards to the role of miRNA in the regulation of PDLSC and SCAP osteo-odontogenic differentiation are shown in Tables 4 and 5, respectively.

**Table 4:** MicroRNAs involved in the regulation of PDLSC osteo-odontogenic differentiation

Effect	MicroRNA	Target (interaction)	Pathway analysis	Significant in vitro effects (assay)	Reference
Differentiation inhibition	miR-584-5p overexpression	H2AFZ (+), RUNX2 (-)	-	Reduced ALP activity (ALPa) Reduced mineralized nodule formation (ARS) Downregulation of differentiation markers: ALPL, SP7, RUNX2 (RT-qPCR)	Wang et al. 2025 [83]
	miR-508-5p overexpression	SOX11 (-)	-	Reduced ALP activity (ALPa) Reduced mineralized nodule formation (ARS) Downregulation of differentiation markers: COL1A1, Runx2, and OCN (RT-qPCR)	Guo et al. 2025 [82]
	miR-142-3p overexpression	SGK1 (-)	-	Reduced ALP activity (ALPa) Reduced mineralized nodule formation (ARS) Downregulation of differentiation markers: Runx2, ALP, OCN (RT-qPCR)	Sun et al. 2023 [77]
	miR-223 overexpression	TGFβR2 (-); FGFR2 (-)	^(A)^MAPK	Reduced mineralized nodule formation (ARS) Downregulation of differentiation markers: Runx2, OPN, OCN (WB)	Zhang et al. 2022 [69]
	miR-200a-3p overexpression	ZEB2 (-)	^(B)^NF-kB	Reduced ALP activity (ALPa) Reduced mineralized nodule formation (ARS) Downregulation of differentiation markers: Runx2, ALP, OCN, OPN (RT-qPCR)	Wang et al. 2022 [76]
	miR-146a overexpression	CELF3 (-)	-	Reduced ALP activity (ALPs; ALPa) Downregulation of differentiation markers: OPN, Runx2, Col1, ALP, OSX, OCN (RT-qPCR)	Meng et al. 2022 [71]
	miR-34a overexpression	CELF3 (-)	-	Reduced ALP activity (ALPs; ALPa) Downregulation of differentiation markers: OPN, Runx2, Col1, ALP, OSX, OCN (RT-qPCR)	Meng et al. 2022 [71]
	miR-452 overexpression	BMI1 (-)	-	Reduced ALP activity (ALPa) Reduced mineralized nodule formation (ARS) Downregulation of differentiation markers: Runx2, OSX, OCN (RT-qPCR)	Mao et al. 2022 [73]
	miR-23b overexpression	Runx2 (-)	^(B)^Wnt/β-catenin	Reduced mineralized nodule formation (ARS) Downregulation of differentiation markers: Runx2 (RT-qPCR; Western blot)	Sun et al. 2021 [67]
	hsa-let-7b overexpression	CTHRC1 (-)	-	Reduced ALP activity (ALPs) Reduced mineralized nodule formation (ARS) Downregulation of differentiation markers: ALP, Runx2, OSX (RT-qPCR; Western blot)	Fu et al. 2021 [62]
	miR-152-3p overexpression	ITGA5 (-)	-	Reduced ALP activity (ALPs) Reduced mineralized nodule formation (ARS) Downregulation of differentiation markers: ALP, Runx2, OCN, Smad4 (RT-qPCR; Western blot)	Wu & Ma 2020 [59]
	miR-375 knockdown	TOB2 (+)	-	Reduced ALP activity (ALPs) Reduced mineralized nodule formation (ARS) Downregulation of differentiation markers: COL1A1, Runx2, OCN (Western blot)	Wang et al. 2020 [60]
	miR-23a overexpression	BMPR1B (-)	^(B)^BMP	Reduced ALP activity (ALPs) Reduced mineralized nodule formation (ARS) Downregulation of differentiation markers: COL1A1, ALP, Runx2, OGN (RT-qPCR)	Zhang et al. 2019 [55]
	miR-132 overexpression	GDF5 (-)	^(B)^NF-kB	Reduced ALP activity (ALPs, ALPa) Reduced mineralized nodule formation (ARS) Downregulation of differentiation markers: ALP, BMP2, Runx2, OCN (RT-qPCR)	Xu et al. 2019 [52]
	miR‐24‐3p overexpression	Smad5 (-)	-	Reduced ALP activity (ALPa) Reduced mineralized nodule formation (ARS) Downregulation of differentiation markers: BMP2, Runx2, OCN (RT-qPCR)	Li et al. 2019 [51]
	miR-22 knockdown	HDAC6 (+)	-	Reduced mineralized nodule formation (ARS) Downregulation of differentiation markers: Runx2, OPN (RT-qPCR; Western blot)	Yan et al. 2017 [46]
	miR-214 overexpression	ATF4 (-)	-	Reduced ALP activity (ALPa) Reduced mineralized nodule formation (ARS) Downregulation of differentiation markers: ALP, Runx2, OCN (RT-qPCR)	Yao et al. 2017 [50]
	miR-21 overexpression	Smad5 (-)	-	Reduced ALP activity (ALPa) Reduced mineralized nodule formation (ARS) Downregulation of differentiation markers: ALP, BSP, Runx2, OSX (RT-qPCR)	Wei et al. 2017 [48]
	miR-374a knockdown	APC (+)	^(B)^Wnt/β-catenin	Reduced mineralized nodule formation (ARS) Downregulation of differentiation markers: ALP, BSP, Runx2, OCN (RT-qPCR)	Cheng et al. 2017 [47]
	miR-214 overexpression	CTNBB1 (-)	^(B)^Wnt/β-catenin	Reduced mineralized nodule formation (ARS) Downregulation of differentiation markers: ALP, BSP, OCN (RT-qPCR)	Cao et al. 2017 [49]
	miR-218 knockdown	SFRP2 (+)	^(B)^Wnt	Reduced mineralized nodule formation (ARS) Downregulation of differentiation markers: ALP, BSP, OCN, Runx2 (RT-qPCR)	Sun et al. 2016 [45]
Differentiation promotion	miR-299-5p overexpression	PUM2 (-)	-	Upregulation of differentiation markers: ALP, Runx2, and OCN (RT-qPCR)	Zhang et al. 2025 [79]
	miR-708-3p overexpression	LSD1 (-)	-	Increased mineralized nodule formation (ARS) Upregulation of differentiation markers: Runx2, and OCN (RT-qPCR)	Shao et al. 2025 [80]
	miR-508-5p knockdown	SOX11 (+)	-	Increased ALP activity (ALPa) Increased mineralized nodule formation (ARS) Upregulation of differentiation markers: COL1A1, Runx2, and OCN (RT-qPCR)	Guo et al. 2025 [82]
	miR-142-3p knockdown	SGK1 (+)	^−^	Increased ALP activity (ALPa) Increased mineralized nodule formation (ARS) Upregulation of differentiation markers: Runx2, ALP, OCN (RT-qPCR)	Sun et al. 2023 [77]
	miR-146a-5p overexpression	TRAF6 (-)	^(A)^NF-kB	Increased expression during osteo/odontogenic differentiation	Yu et al. 2022 [70]
	miR-200a-3p knockdown	ZEB2 (+)	^(B)^NF-kB	Increased ALP activity (ALPa) Increased mineralized nodule formation (ARS) Upregulation of differentiation markers: Runx2, ALP, OCN, OPN (RT-qPCR)	Wang et al. 2022 [76]
	miR‑589‑3p overexpression	ATF1 (-)	^(A)^Wnt; MAPK	Increased ALP activity (ALPs) Increased mineralized nodule formation (ARS) Upregulation of differentiation markers: Runx2, OCN, OSX (Western blot)	Shi et al. 2022 [74]
	miR-383-5p overexpression	HDAC9 (-)	-	Increased ALP activity (ALPs) Increased mineralized nodule formation (ARS) Upregulation of differentiation markers: Runx2, OCN, Smad4 (RT-qPCR; western blot)	Ma & Wu 2021 [66]
	miR-30a overexpression	CTSK (+)	^(B)^Wnt/β-catenin	Upregulation of differentiation markers: CAP, CEMP (Immunohistochemistry)	Liu et al. 2021 [65]
	miR-153-3p knockdown	KDM6A (+)	^−^	Increased ALP activity (ALPs) Increased mineralized nodule formation (ARS) Upregulation of differentiation markers: ALP, Runx2, OPN (RT-qPCR; Western blot)	Jiang & Jia 2021 [64]
	miR-155-5p knockdown	Est1	-	Increased ALP activity (ALPs) Increased mineralized nodule formation (ARS) Upregulation of differentiation markers: COL-1, Runx2, OCN (RT-qPCR; Western blot)	Hua & Zhang 2021 [68]
	hsa-let-7b knockdown	CTHRC1 (+)	-	Increased ALP activity (ALPs) Increased mineralized nodule formation (ARS) Upregulation of differentiation markers: ALP, Runx2, OSX (RT-qPCR; Western blot)	Fu et al. 2021 [62]
	miR-152-3p knockdown	ITGA5 (+)	-	Increased ALP activity (ALPs) Increased mineralized nodule formation (ARS) Upregulation of differentiation markers: ALP, Runx2, OCN, Smad4 (RT-qPCR; Western blot)	Wu & Ma 2020 [59]
	miR-4262 knockdown	SOCS4 (+)	-	Increased ALP activity (ALPs) Increased mineralized nodule formation (ARS) Upregulation of differentiation markers: Runx2, OPG, COL1A1 (RT-qPCR; Western blot)	Wei et al. 2020 [58]
	miR-375 overexpression	TOB2 (-)	-	Increased ALP activity (ALPs) Increased mineralized nodule formation (ARS) Upregulation of differentiation markers: COL1A1, Runx2, OCN (Western blot)	Wang et al. 2020 [60]
	miR-132 knockdown	GDF5 (+)	^(B)^NF-kB	Increased ALP activity (ALPs, ALPa) Increased mineralized nodule formation (ARS) Upregulation of differentiation markers: ALP, BMP2, Runx2, OCN (RT-qPCR)	Xu et al. 2019 [52]
	miR‐24‐3p knockdown	Smad5 (+)	-	Increased ALP activity (ALPa) Increased mineralized nodule formation (ARS) Upregulation of differentiation markers: BMP2, Runx2, OCN (RT-qPCR)	Li et al. 2019 [51]
	miR-148a knockdown	NRP1 (+)	-	Increased ALP activity (ALPs, ALPa) Increased mineralized nodule formation (ARS) Upregulation of differentiation markers: ALP, Runx2, OCN (RT-qPCR)	Bao et al. 2019 [54]
	miR-22 overexpression	HDAC6 (-)	-	Increased mineralized nodule formation (ARS) Upregulation of differentiation markers: Runx2, OPN (RT-qPCR; Western blot)	Yan et al. 2017 [46]
	miR-21 knockdown	Smad5 (+)	-	Increased ALP activity (ALPa) Increased mineralized nodule formation (ARS) Upregulation of differentiation markers: ALP, BSP, Runx2, OSX (RT-qPCR)	Wei et al. 2017 [48]
	miR-374a overexpression	APC (-)	^(B)^Wnt/β-catenin	Increased mineralized nodule formation (ARS) Upregulation of differentiation markers: ALP, BSP, Runx2, OCN (RT-qPCR)	Cheng et al. 2017 [47]
	miR-214 knockdown	CTNBB1 (+)	^(B)^Wnt/β-catenin	Increased mineralized nodule formation (ARS) Upregulation of differentiation markers: ALP, BSP, OCN (RT-qPCR)	Cao et al. 2017 [49]
	miR-218 overexpression	SFRP2 (-)	^(B)^Wnt	Increased mineralized nodule formation (ARS) Upregulation of differentiation markers: ALP, BSP, OCN, Runx2 (RT-qPCR)	Sun et al. 2016 [45]

(+): miRNA increases target activity (upregulation/enhancement); (-): miRNA decreases target activity (downregulation/repression). (A) Obtained through bioinformatic analysis; (B) Tested via western blot analysis.

**Table 5:** MicroRNAs involved in the regulation of SCAP osteo-odontogenic differentiation

Effect	MicroRNA	Target	Pathway analysis	Significant in vitro effect	Reference
Differentiation inhibition	miR-143-3p overexpression	NFIC (-)	-	Reduced mineralized nodule formation (ARS) Downregulation of differentiation markers: NFIC, DSP, DSPP, KLF4 (RT-qPCR)	Gao et al. 2022 [75]
	miR-497-5p knockdown	Smurf2 (-)	^(B)^Smad	Reduced ALP activity (ALPs; ALPa) Reduced mineralized nodule formation (ARS) Downregulation of differentiation markers: DSPP, COL1, ALP, Runx2, OSX (RT-qPCR)	Liu et al. 2020 [61]
Differentiation promotion	miR-146a-5p overexpression	TRAF6 (-)	^(A)^NF-kB	Increased expression during osteo/odontogenic differentiation	Yu et al. 2022 [70]
	miR-497-5p overexpression	Smurf2	^(B)^ TGF-β Smad	Increased ALP activity (ALPs; ALPa) Increased mineralized nodule formation (ARS) Upregulation of differentiation markers: DSPP, COL1, ALP, Runx2, OSX, OPN (Western blot)	Chen et al. 2022 [72]
	miR-497-5p overexpression	Smurf2 (-)	^(B)^Smad	Increased ALP activity (ALPs; ALPa) Increased mineralized nodule formation (ARS) Upregulation of differentiation markers: DSPP, COL1, ALP, Runx2, OSX (RT-qPCR)	Liu et al. 2020 [61]
	miR-34a overexpression	NOTCH2 (-)	^(B)^Notch	Increased ALP activity (ALPs) Increased mineralized nodule formation (ARS) Upregulation of differentiation markers: DSPP, OCN, Runx2, OSX (RT-qPCR)	Sun et al. 2014 [44]
	miR-615-3p knockdown	PVT1 (+)	^−^	Increased ALP activity (ALPs; ALPa) Increased mineralized nodule formation (ARS) Upregulation of differentiation markers: DSPP, DMP1 (qPCR, immunofluorescence staining, Western blot)	Yang et al. 2025 [81]

(+): miRNA increases target activity (upregulation/enhancement); (-): miRNA decreases target activity (downregulation/repression). (A) Obtained through bioinformatic analysis; (B) Tested via western blot analysis

The overexpression of miR-584-5p, miR-508-5p, miR-142-3p, miR-223, miR-200a-3p, miR-34a, miR-452, miR-23b, hsa-let-7b, miR-152-3p, miR-23a, miR-132, miR‐24‐3p, miR-214, miR-21 and miR-214, and the knockdown of miR-375, miR-22, miR-374a and miR-218 resulted in a reduced PDLSC osteo-odontogenic differentiation. Contrarily, an increased PDLSC osteo-odontogenic differentiation was observed with the overexpression of miR-146a-5p, miR‑589‑3p, miR-383-5p, mir-30a, hsa-let-7b, miR-375, miR-22, miR-374a, miR-218 and the knockdown of miR-508-5p, miR-142-3p, miR-200a-3p, miR-153-3p, miR-155-5P, miR-152-3p, miR-132, miR‐24‐3p, miR-21 and miR-214. In an inflammatory microenvironment (porphyromonas gingivalis LPS or TNF-α induced), the overexpression of miR-299-5p and miR-708-3p, and the knockdown of miR-4262 and miR-148a resulted in an increased PDLSC osteo-odontogenic differentiation.

The overexpression of miR-143-3p and the knockdown of miR-497-5p resulted in a reduced SCAP osteo-odontogenic differentiation. Contrarily, an increased SCAP osteo-odontogenic differentiation was observed with the overexpression of miR-146a-5p, miR-497-5p and miR-34a, and the knockdown of miR-615-3p.

#### Influence of MiRNA on Cell Inflammation

The extracted data from the selected studies with regards to the role of miRNA in the regulation of PDLSC inflammation are shown in Table 6.

**Table 6:** MicroRNAs involved in the regulation of PDLSC inflammation

MicroRNA	PDLSC stimuli	Target	Function	Significant in vitro effect (assay)	Reference
miR-299-5p overexpression	Inflammation (Lipopolysaccharides)	PUM2 (-)	Reduces inflammation	Reduced IL-6, IL-1β, and TNF-α expression (ELISA)	Zhang et al. 2025 [79]
miR-671-5p knockdown	Inflammation (Lipopolysaccharides)	DUSP8 (+)	Reduces inflammation	Reduced IL-6, IL-1β, and TNF-α expression (RT-qPCR; ELISA)	Wang et al. 2024 [78]
miR-146a overexpression	-	-	Reduces inflammation	Reduced IL-13 expression (RT-qPCR; Western Blot)	Wang et al. 2021 [63]
miR-4262 knockdown	Inflammation (TNF-α)	SOCS4 (+)	Reduces inflammation	Reduced IL-1β, IL-6, and MCP-1 expression (ELISA)	Wei et al. 2020 [58]
miR‐146a overexpression	-	-	Reduces inflammation	Reduced IL-17 and IL-35 expression (Western Blot)	Zhao et al. 2019 [53]

The overexpression of miR-299-5p and miR‐146a, and the knockdown of miR-671-5p and miR-4262 resulted in a reduced PDLSC inflammatory marker expression.

### Quality Assessment

The results of the quality assessment of the included studies using our previously mentioned miRoB-DSC tool are presented in Table 7.

**Table 7:** Quality assessment results

Study	Items from the miRoB-DSC tool																	
	1	2	3	4	5	6	7	8	9	10	11	12	13	14	15	16	17	Total (%)
Sun et al., 2014 [44]	Y	Y	N	Y	Y	Y	N	Y	Y	N	Y	Y	Y	Y	Y	N	Y	13 (76,5)
Sun et al., 2016 [45]	Y	Y	N	Y	Y	Y	N	Y	Y	N	Y	Y	Y	Y	Y	N	N	12 (70,6)
Bao et al., 2019 [54]	Y	Y	Y	Y	Y	Y	N	Y	Y	N	Y	Y	Y	Y	Y	N	N	13 (76,5)
Zhang et al., 2019 [55]	Y	Y	Y	Y	Y	Y	N	Y	Y	N	Y	Y	Y	Y	Y	N	N	13 (76,5)
Li et al., 2019 [51]	Y	Y	Y	Y	Y	Y	N	Y	Y	N	Y	Y	Y	Y	Y	N	N	13 (76,5)
Liu et al., 2020 [57]	Y	Y	Y	Y	Y	Y	N	Y	Y	N	Y	Y	Y	Y	Y	Y	Y	15 (88,2)
Wei et al., 2020 [58]	N	N	N	Y	Y	N	N	Y	Y	N	Y	Y	Y	Y	Y	N	N	9 (52,9)
Wu et al., 2020 [59]	Y	N	Y	Y	Y	Y	N	Y	Y	N	Y	Y	Y	Y	Y	N	Y	13 (76,5)
Wang et al., 2020 [60]	Y	Y	Y	Y	Y	Y	N	Y	Y	N	Y	Y	Y	Y	Y	N	Y	14 (82,4)
Liu et al., 2020 [61]	Y	Y	N	Y	Y	Y	N	Y	Y	N	Y	Y	Y	Y	Y	N	N	12 (70,6)
Fu et al., 2021 [62]	Y	Y	Y	Y	Y	Y	N	Y	Y	N	Y	Y	Y	Y	Y	N	N	13 (76,5)
Wang et al., 2021 [63]	Y	Y	N	Y	Y	Y	N	Y	Y	N	Y	Y	Y	Y	Y	N	N	12 (70,6)
Yan et al., 2017 [46]	Y	N	Y	Y	Y	Y	N	Y	Y	N	Y	Y	Y	Y	Y	N	N	12 (70,6)
Jiang et al., 2021 [64]	Y	Y	Y	Y	Y	Y	N	Y	Y	N	Y	Y	Y	Y	Y	N	N	13 (76,5)
Liu et al., 2021 [65]	Y	Y	Y	Y	Y	Y	N	Y	Y	N	Y	Y	Y	Y	Y	N	Y	14 (82,4)
Ma et al., 2021 [66]	Y	Y	Y	Y	Y	Y	N	Y	Y	N	Y	Y	Y	Y	Y	N	Y	14 (82,4)
Sun et al., 2021 [67]	Y	Y	Y	Y	Y	Y	N	Y	Y	N	Y	Y	Y	Y	Y	N	N	13 (76,5)
Hua et al., 2021 [68]	Y	Y	Y	Y	Y	Y	N	Y	Y	N	Y	Y	Y	Y	Y	N	N	13 (76,5)
Zhang et al., 2022 [69]	Y	Y	Y	Y	Y	Y	N	Y	Y	N	Y	Y	Y	Y	Y	Y	Y	15 (88,2)
Yu et al., 2022 [70]	-	-	-	Y	Y	N	N	-	Y	N	Y	Y	Y	Y	Y	Y	Y	10 (76,9)
Meng et al., 2022 [71]	Y	Y	Y	Y	Y	Y	N	Y	Y	N	Y	Y	Y	Y	Y	N	Y	14 (82,4)
Chen et al., 2022 [72]	Y	N	N	Y	Y	Y	N	Y	Y	N	Y	Y	Y	Y	Y	N	N	11 (64,7)
Mao et al., 2022 [73]	Y	N	Y	Y	Y	Y	N	Y	Y	N	Y	Y	Y	Y	Y	N	N	12 (70,6)
Cheng et al., 2017 [47]	Y	N	Y	Y	Y	Y	N	Y	Y	N	Y	Y	Y	Y	Y	N	N	12 (70,6)
Shi et al., 2022 [74]	N	N	Y	Y	Y	Y	N	Y	Y	N	Y	Y	Y	Y	Y	Y	Y	13 (76,5)
Gao et al., 2022 [75]	Y	Y	Y	Y	Y	Y	N	Y	Y	N	Y	Y	Y	Y	Y	Y	N	14 (82,4)
Wang et al., 2022 [76]	Y	N	Y	Y	Y	Y	N	Y	Y	N	Y	Y	Y	Y	Y	N	Y	13 (76,5)
Sun et al., 2023 [77]	Y	Y	Y	Y	Y	Y	N	Y	Y	N	Y	Y	Y	Y	Y	N	Y	14 (82,4)
Wang et al., 2024 [78]	Y	Y	Y	Y	Y	Y	N	Y	Y	N	Y	Y	Y	Y	Y	Y	N	14 (82,4)
Zhang et al., 2025 [79]	N	N	Y	Y	Y	Y	N	Y	Y	N	Y	Y	Y	Y	Y	Y	Y	13 (76,5)
Shao et al., 2025 [80]	Y	Y	Y	Y	Y	Y	N	Y	Y	N	Y	Y	Y	Y	Y	N	N	13 (76,5)
Yang et al., 2025 [81]	Y	Y	Y	Y	Y	Y	N	Y	Y	N	Y	Y	Y	Y	Y	N	Y	14 (82,4)
Guo et al., 2025 [82]	Y	Y	Y	Y	Y	Y	N	Y	Y	N	Y	Y	Y	Y	Y	N	N	13 (76,5)
Wei et al., 2017 [48]	Y	N	Y	Y	Y	Y	N	Y	Y	N	Y	Y	Y	Y	Y	Y	N	13 (76,5)
Cao et al., 2017 [49]	Y	Y	Y	Y	Y	Y	N	Y	Y	N	Y	Y	Y	Y	Y	N	N	13 (76,5)
Yao et al., 2017 [50]	Y	Y	Y	Y	Y	Y	N	Y	Y	N	Y	Y	Y	Y	Y	N	N	13 (76,5)
Li et al., 2019 [56]	Y	Y	Y	Y	Y	Y	N	Y	Y	N	Y	Y	Y	Y	Y	N	N	13 (76,5)
Xu et al., 2019 [52]	Y	Y	Y	Y	Y	Y	N	Y	Y	N	Y	Y	Y	Y	Y	N	N	13 (76,5)
Zhao et al., 2019 [53]	Y	N	N	Y	Y	N	N	Y	Y	N	Y	Y	Y	Y	Y	N	N	10 (58,8)
Wang 2025 [83]	Y	Y	Y	Y	Y	Y	N	Y	Y	N	Y	Y	Y	Y	Y	N	N	13 (76,5)

-: not applicable

Item fulfillment ranged from 15/17 to 10/17. Most of the studies fulfilled 13/17 items. Items 7 and 10 regarding random allocation and blinded outcome assessment were not fulfilled by any of the studies. If discarded from the quality assessment analysis, most of the studies present more than 80% of item fulfillment. These results indicate a high transparency and replicability.

## Discussion

### On the Studies’ Methodology

Across the included studies, the typical workflow to test how microRNAs modulate DSC behavior followed a common, reproducible arc: 1) cell sourcing and characterization, 2) miRNA gain/loss-of-function with pathway “rescue”, 3) functional readouts of osteo-odontogenic differentiation and cell behavior, and 4) mechanistic target validation. Human PDLSCs were usually isolated from premolars/third molars of healthy orthodontic donors (often early passages, cultured in α-MEM/DMEM + 10% FBS) and phenotyped by colony formation and MSC markers (e.g., STRO-1, CD90, CD105, CD146; negatives CD34/CD45); while SCAPs were obtained from apical papilla of immature teeth and similarly validated (e.g., CD24/STRO-1, multipotency) [44, 48, 50, 52, 56, 60–62, 66, 67, 75, 82]. Two studies used commercially-available DSCs [74, 79]. Perturbation of miRNA levels relied on synthetic mimics/inhibitors or lentiviral vectors, with parallel negative controls and, critically, rescue experiments by overexpressing or silencing the predicted target gene to reverse the miRNA effect [46, 48, 49, 51, 52, 60, 66, 76, 82]. Complementarily, various studies used groups that modeled disease-relevant contexts such as inflammatory cues (using LPS or TNF-α) [54, 58, 79, 80].

Functional outcomes were consistently evaluated with a shared toolkit that combined molecular, biochemical, and phenotypic readouts. At the molecular level, qRT-PCR and western blotting were employed to quantify osteo-odontogenic markers such as RUNX2, ALP, OCN, DSPP, and DMP1, providing both transcriptional and translational resolution of lineage commitment. These were majorly complemented by enzymatic and mineralization assays, including ALP activity/colorimetric staining and Alizarin Red S detection of calcium-rich deposits, with spectrophotometric quantification to standardize results across studies [44–83]. Many studies also assessed cellular behaviors critical for regenerative potential, such as cell viability and proliferation (CCK-8 metabolic assays or MTT assays) [53, 57, 59, 60, 63, 74, 77, 78]. Where inflammatory or microenvironmental modulation was under investigation, cytokine and chemokine secretion was profiled via ELISA panels or Western Blot, linking miRNA regulation to immunomodulatory signaling [53, 54, 58, 63, 78–80].

Mechanistic validation of predicted miRNA–mRNA interactions followed a similar approach across studies. After initial computational target prediction (usually via TargetScan, miRanda, or miRDB), most studies employed a dual-luciferase reporter assay to confirm direct binding. In this method, the wild-type (WT) 3′ untranslated region (3′UTR) of the candidate target gene is cloned downstream of a luciferase reporter, while a mutant (MUT) construct carries point substitutions in the predicted miRNA seed-binding site. Co-transfection with miRNA mimics or inhibitors then reveals whether luciferase activity is repressed in the WT but not the MUT construct, thus providing functional evidence of direct targeting [48, 50–52, 66, 75, 80, 82].

Lastly, some studies extended beyond target validation to pathway-level interrogation, with the assessment of several canonical signaling cascades. The most frequently examined was Wnt/β-catenin signaling, assessed by TOP/FOP-Flash luciferase reporter assays in combination with western blot analysis of β-catenin and validation of direct miRNA targets [47, 49], by evaluating β-catenin and p-GSK3β expression and pharmacological modulation with a Wnt agonist [67], indirectly through targeting of Wnt antagonists without direct measurement of β-catenin activity [45], and through KEGG pathway enrichment analysis [74]. TGF-β/Smad signaling was assessed by evaluating Smad2, Smad3, and Smad4 expression in response to miRNA modulation, supported by luciferase validation of Smurf2 as a direct target and rescue experiments involving Smurf2 silencing [61, 72]. NF-κB signaling was assessed by measuring phosphorylation of p65 and IκBα in combination with miRNA gain- and loss-of-function and rescue experiments [52], by examining p65 phosphorylation and nuclear translocation following miRNA modulation [76], and through KEGG pathway enrichment [70]. NOTCH signaling was investigated through combined expression profiling and functional modulation, including RT-PCR and immunofluorescence of pathway components, ligand- and inhibitor-based activation or suppression (JAG1 and DAPT), assessment of HES1 expression and NOTCH2 intracellular domain nuclear localization, and dual-luciferase reporter assays validating direct miRNA–NOTCH interactions [44]. BMP signaling was assessed by evaluating Smad1/5/9 phosphorylation in response to miRNA modulation, supported by luciferase validation of BMPR1B as a direct target and rescue experiments restoring BMP pathway activity [55]. In contrast, MAPK pathway involvement was inferred solely through bioinformatic analyses, primarily KEGG pathway enrichment of predicted miRNA target genes, without direct experimental interrogation of MAPK signaling activity [69, 74, 77].

### On the Studies’ Results

To our knowledge, this is the first systematic review to assess the influence of miRNA on the viability, proliferation, osteo-odontogenic differentiation potential and/or inflammation of PDLSCs and SCAPs. Nevertheless, there are previous relevant reviews on the field that should be discussed. Collectively, three reviews have analyzed microRNA regulation of dental pulp stem cells (DPSCs), reporting a wide range of molecules with either promotive or inhibitory influence on odontogenic and osteogenic differentiation. Reported pro-differentiation miRNAs include miR-27a-5p, miR-125a-3p, miR-146a-5p, miR-223, miR-675, miR-720, miR-3065, miR-216a, miR-543, miR-223-3p, miR-21, miR-25-5p, miR-633, miR-24-3p, miR-196a, and miR-188-3p, which were shown to enhance mineralization and increase the expression of markers such as RUNX2, ALP, DSPP, and DMP-1 through pathways including TGFβ/Smad, NOTCH1, and STAT3. By contrast, anti-differentiation miRNAs identified across the reviews comprise miR-135b, miR-140-5p, miR-143, miR-143-5p, miR-215, miR-219a-1-3p, miR-295-5p, miR-488, miR-24-3p, miR-30b-3p, miR-188-3p, miR-496, miR-617, miR-588, miR-143-3p, miR-206, miR-31, miR-3658, miR-508-5p, hsa-let-7c, miR-218, and miR-145, which were reported to suppress lineage commitment through inhibition of key transcription factors or signaling pathways such as RUNX2, MAPK, Wnt/β-catenin, NF-κB [84–86].

**Fig. 2:**
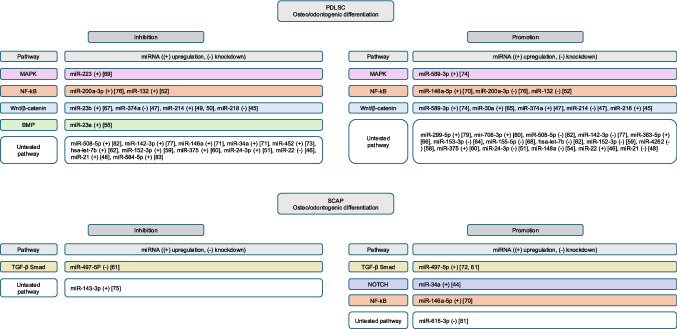
Summary of the miRNAs found to promote or inhibit PDLSC and SCAP osteo-odontogenic differentiation and their implicated pathways

An illustrative summary of the miRNAs identified in the present systematic review that promote or inhibit PDLSC and SCAP osteo-odontogenic differentiation and their implicated pathways, if tested by the included studies, is shown in Fig. 2. When cross-referencing data from the three reviews on miRNAs and DPSCs with data from the present review regarding PDLSCs and SCAPs, a limited shared set emerges with both conserved and niche-specific directions. miR-146a-5p appears a notable constant, promoting differentiation in all three cell types [70, 84–86]. In contrast, miR-34a shows opposite behavior, promoting SCAP differentiation [44] but inhibiting that of PDLSCs [71]. Several miRNAs exhibit context-dependent switching between niches: miR-21 and miR-223 promote DPSC differentiation [84–86] yet suppress PDLSCs [48, 69]; miR-218 inhibits DPSC differentiation [84–86] but promotes PDLSCs [45]; and miR-24-3p is dual within DPSCs while inhibitory in PDLSCs [51]. miR-508-5p inhibits DPSC differentiation [84–86] but is study-dependent in PDLSCs (reported as both inhibitory and promotive) [82]. By comparison, miR-143-3p behaves as a consistent inhibitor in both DPSCs and SCAPs. Overall, these overlaps support a partially shared miRNA regulation, although directionality may be dependent on niche and context [84–86].

Other miRNAs reported may reflect either niche-specific biology or simply a lack of studies in other DSCs to date. These are, in PDLSCs: miR-584-5p, miR-23a/b, miR-132, miR-142-3p, miR-152-3p, miR-200a-3p, miR-299-5p, miR-374a, miR-375, miR-383-5p, miR-452, miR-589-3p, miR-708-3p, and miR-30a, which have not yet been reported in DPSCs or SCAPs. Similarly, miR-497-5p has only been described in SCAPs, where it promotes differentiation. By contrast, several miRNAs identified in DPSCs, such as miR-675, miR-720, miR-3065, miR-216a, miR-543, miR-25-5p, miR-633, and miR-196a on the promotive side, and miR-135b, miR-140-5p, miR-143-5p, miR-215, miR-219a-1-3p, miR-295-5p, miR-488, miR-30b-3p, miR-496, miR-617, miR-588, miR-206, miR-31, miR-3658, and miR-145 as inhibitors, have not been reported in PDLSCs or SCAPs to date. Taken together, these comparisons reinforce that while a core subset of miRNAs appears central to osteo-odontogenic differentiation across dental stem cell types, functional outcomes can diverge according to cell origin and experimental context, and the current evidence base remains uneven across niches.

Comparison of the signaling pathways identified in the three DPSC reviews with those reported in the present review of PDLSCs and SCAPs reveals both convergence on common regulatory axes and divergence in the specific effectors studied. In all tested DSC niches, miRNAs were found to converge on core differentiation pathways, most notably RUNX2 regulation (targeted by miR-23b [67] in PDLSCs, and by miR-218 in DPSCs [84–86]), Smad/TGFβ signaling (miR-497-5p [61, 72] in PDLSCs versus miR-223 and miR-24-3p in DPSCs [84–86]), NOTCH signaling (miR-34a [44] targeting NOTCH2 in SCAPs and miR-146a-5p targeting NOTCH1 in DPSCs[84–86]), NF-κB regulation (miR-146a-5p [70], miR-132 [52] and miR-200a-3p [76] in PDLSCs, miR-125a-3p and miR-143 in DPSCs [84–86]), and Wnt/β-catenin signaling (multiple regulators in PDLSCs including miR‑589‑3p [74], miR-23b [67], miR-374a [47], miR-214 [49], miR-218 [45] compared with miR-140-5p in DPSCs [84–86]). Lastly, via bioinfomatic analyses, various studies highlighted the possible implication of MAPK signaling in miRNA regulation of the osteo/odontogenic differentiation of PDLSCs (miR-223 [69] and miR‑589‑3p [74]). In this regard, two regulators have been identified targeting MAPK1 in DPSCs (miR-143-5p, miR-488 m, and miR-218 [84, 85]).

A previous narrative review synthesized evidence on non-coding RNAs, including microRNAs, long non-coding RNAs, and circular RNAs, in the osteogenic differentiation of PDLSCs [87]. Consistent with our data, his study also highlighted several miRNAs implicated in the osteogenic differentiation of PDLSCs, notably miR-21, miR-146a, the miR-17–92 cluster, miR-214, and miR-125b. Nevertheless, their review did not follow a systematic methodology, nor did it incorporate a structured assessment of study quality. Similarly, a previous scoping review synthesized miRNAs regulating the osteogenic differentiation of PDLSCs [20]. In line with our findings, both reviews converge on miR-22, miR-374a, miR-383-5p, miR-589-3p, miR-214, miR-24-3p, miR-152-3p, miR-218, let-7b, and miR-21 as recurrent regulators. Of these, miR-22, miR-374a, miR-383-5p, and miR-589-3p were consistently reported as promotive, while miR-214, miR-24-3p, miR-152-3p, and miR-218 were predominantly inhibitory. The earlier work also emphasized miRNAs not retrieved in our dataset, including miR-758, miR-101, miR-181b-5p, miR-543, miR-2861, miR-17, miR-7, miR-106a-5p, miR-222-3p, miR-4781-3p, miR-125b, miR-153-3p, miR-10a-5p, miR-30c, miR-155-5p, miR-184, and miR-874-3p. Conversely, our review uniquely identified miR-299-5p, miR-708-3p, miR-508-5p, miR-146a/miR-146a-5p, miR-30a, miR-375, miR-142-3p, miR-223, miR-200a-3p, miR-34a, miR-452, miR-23a/b, and miR-132, which were not discussed in their analysis. Taken together, these complementary observations indicate a core subset of overlapping miRNAs while also expanding the regulatory landscape with niche- or context-specific candidates. Importantly, some regulators such as miR-21, let-7b, and miR-218 displayed divergent roles between the two reviews, underlining the context-dependency of miRNA function across experimental settings.

Lastly, regarding the influence of inflammation on the relationship between miRNA and PDLSCs, the same study identified a series of miRNA which, under inflammatory condition such as periodontitis, force induction, diabetes or smoking, promote (miR-17, miR-21, miR-146a, miR-3679-3p, miR-6747-5p) or suppress (miR-23a, miR-23b, miR-27a-3p, miR-138, miR-182, miR-148a, miR-195-5p, miR-31, miR-1305) osteogenic differentiation [20]. The results from our systematic review identify four additional miRNAs which have been found to promote PDLSC osteo/odontogenic differentiation when overexpressed (miR-299-5p [79], miR-708-3p [80]) or inhibited (miR-4262 [58], miR-148a [54]) under LPS or TNFα-mediated inflammation. Additionally, in the present review, five microRNA were found to reduce inflammatory markers from PDLSCs when overexpressed (miR-299-5p [79], miR-146a [53, 63]) or inhibited (miR-671-5p [78], miR-4262 [58]).

### Limitations

This systematic review has several limitations that should be considered when interpreting its findings. First, all included studies were in vitro experiments, which inherently limits the direct extrapolation of results to in vivo conditions or clinical applications. While in vitro models are indispensable for mechanistic investigation of microRNA–target interactions and signaling pathways, they cannot fully recapitulate the complex cellular, vascular, immune, and mechanical microenvironments present in periodontal and periapical tissues. Consequently, the functional effects of specific microRNAs on PDLSC and SCAP behavior observed in controlled culture conditions may differ in magnitude or even direction in vivo, particularly under disease-relevant conditions such as chronic inflammation or impaired healing.

Secondly, substantial methodological heterogeneity was observed across the included studies, encompassing cell sources (donor age, tooth type, health status), culture conditions, miRNA manipulation strategies (mimics, inhibitors, viral vectors), outcome measures, and duration of differentiation protocols. This heterogeneity precluded quantitative synthesis and meta-analysis and required a narrative approach to data integration.

In addition, a specific tailor-made quality assessment tool was used in this systematic review. The miRoB-DSC is a newly developed tool and was not specifically validated before its use in this study. However, its effectiveness is supported by its grounding in previously validated in vitro quality assessment frameworks and by its consistent performance when applied to the included studies. The domains and items of miRoB-DSC were adapted from established tools such as ToxRTool [42] and the QUIN tool [43], which have been widely used to assess reliability and reporting quality in experimental in vitro research. When applied to the studies included in this review, miRoB-DSC was able to discriminate between levels of methodological transparency, yielding a broad range of fulfillment scores and consistently identifying known limitations of dental stem cell miRNA studies, such as the absence of randomization and blinded outcome assessment. Importantly, studies with more detailed reporting of cell characterization, experimental controls, and statistical analyses tended to achieve higher fulfillment percentages, supporting the construct validity and practical applicability of the tool within this evidence base.

Lastly, the evidence base remains uneven across dental stem cell niches and biological contexts. Compared with PDLSCs, relatively few studies investigated SCAPs, and only a minority of experiments assessed microRNA effects under inflammatory or disease-mimicking conditions. Moreover, pathway involvement was not uniformly validated across studies, with some signaling cascades inferred solely through bioinformatic enrichment rather than direct functional interrogation. As a result, the regulatory networks identified in this review should be interpreted as context-dependent and provisional, underscoring the need for standardized experimental protocols, broader validation across stem cell populations, and well-designed in vivo studies to strengthen translational relevance.

### Implications for Future Research

Future studies should aim to integrate high-throughput approaches such as next-generation sequencing, multi-omics profiling, and single-cell analyses to obtain a more comprehensive map of the miRNA-mRNA interaction landscape in DSCs [88, 89]. Functional studies in relevant preclinical models and clinical trials will be required to validate candidate miRNAs as therapeutic targets or biomarkers for regenerative applications. Particular attention should also be given to the role of exosomal miRNAs [16], the influence of microenvironmental conditions such as inflammation [90], and the potential synergy between miRNAs and biomaterial-based delivery systems [91]. Collectively, these directions may accelerate the translation of miRNA-based strategies into predictable and safe biologically based therapies in Endodontics and Periodontology.

### Clinical Translation

From a translational perspective, the findings of this systematic review suggest that microRNAs represent promising biological modulators for future regenerative strategies in both endodontics and periodontology, although their clinical application remains preliminary. In the endodontic field, the identification of miRNAs that regulate SCAP osteo-odontogenic differentiation and inflammatory responses supports their potential role in enhancing regenerative endodontic treatments, where SCAP survival, differentiation, and immunomodulation are critical determinants of continued root development and tissue repair [92]. Similarly, in periodontology, miRNA-mediated regulation of PDLSC differentiation and inflammatory signaling highlights a possible approach to improve periodontal regeneration by enhancing host responses rather than relying on cell transplantation. Importantly, these findings align with the growing interest in cell-free approaches, such as the use extracellular vesicles or miRNA-enriched biomaterials, which may offer greater safety, standardization, and regulatory feasibility than direct stem cell therapies [93, 94].

Nevertheless, the exclusively in vitro nature of the current evidence, the heterogeneity of experimental conditions, and the limited evaluation under disease-relevant environments underscore that miRNAs should presently be regarded as biological targets or adjunctive modulators rather than ready-to-use clinical agents. Carefully designed in vivo studies and translational models are therefore essential before miRNA-based interventions can be meaningfully integrated into regenerative endodontic or periodontal protocols.

## Conclusions

This systematic review is the first to comprehensively evaluate the influence of miRNAs on the behavior PDLSCs and SCAPs. miRNA participate in the regulation of DSC osteo-odontogenic differentiation, proliferation and inflammation. Comparisons with previous reviews on DPSCs and PDLSCs suggest both shared and niche-specific regulatory networks. Various signaling pathways have been majorly implicated with miRNA regulation, including RUNX2, Smad/TGFβ, NOTCH, NF-κB, Wnt/β-catenin and MAPK. Overall, miRNAs emerge as promising modulators and potential therapeutic targets in periodontal and endodontic regeneration.

## Supplementary Information

Below is the link to the electronic supplementary material.

Supplementary file1 (DOCX 28 KB)

## Data Availability

All data generated or analysed during this study are included in this published article [and its supplementary information files].
