# Supplementary material for: The Role of MicroRNA in the Behaviour of Periodontal Ligament Stem Cells and Stem Cells from the Apical Papilla: A Systematic Review
Source: Stem Cell Rev Rep. 2026 Mar 12;22(4):1712–38. doi: 10.1007/s12015-026-11088-7 (PMC13100015; doi:10.1007/s12015-026-11088-7)
Supplement: Supplementary file 1 — Supplementary file1 (DOCX 28 KB) [file 12015_2026_11088_MOESM1_ESM.docx]

**miRoB-DSC**

**(MicroRNA Risk of Bias tool for Dental Stem Cell studies)**

**Checklist**

| **Item n** | **Item question** | **Fulfillment (Y/N)** |
| --- | --- | --- |
| **Domain 1. Cell Source and Characterization** | | |
| **1** | Was the origin of the cells clearly described (tooth type, donor age/health, tissue source)? |  |
| **2** | Were cell identity and stemness markers verified (e.g., MSC markers, SCAP-specific CD24)? |  |
| **3** | Were passage number and culture conditions reported? |  |
| **Domain 2. Experimental Design** | | |
| **4** | Were appropriate controls included (e.g., negative/positive controls)? |  |
| **5** | Were interventions (miRNA overexpression/inhibition) adequately described and validated? |  |
| **6** | Were sufficient biological replicates used (≥3 donors or independent experiments)? |  |
| **7** | Were treatment groups randomly assigned? |  |
| **8** | Were culture conditions standardized across groups? |  |
| **Domain 3. Outcome Assessment** | | |
| **9** | Were relevant and validated outcome measures used (e.g., qPCR, Western blot, ALP, ARS staining, mineralization assays)? |  |
| **10** | Were outcome assessors blinded or were objective, automated methods applied? |  |
| **11** | Was normalization to housekeeping genes/proteins appropriately performed? |  |
| **Domain 4. Data Reporting and Analysis** | | |
| **12** | Were statistical methods clearly described and appropriate for the design? |  |
| **13** | Were effect sizes and variability measures (e.g., SD/SEM) reported? |  |
| **14** | Was selective outcome reporting avoided (all planned outcomes reported)? |  |
| **Domain 5. Risk of Bias and Reproducibility** | | |
| **15** | Were conflicts of interest declared, and funding sources disclosed? |  |
| **16** | Were raw data or supplementary datasets made available? |  |
| **17** | Were potential sources of bias and limitations discussed? |  |

Y: fulfilled; N: non-fulfilled

**Item explanation and elaboration**

**Domain 1. Cell Source and Characterization**

Item 1. Cell origin

- Was the source of the dental tissue clearly described (e.g., tooth type: third molar, immature incisor, premolar; human vs animal)?
- Was donor information provided (species, age, health status, presence/absence of inflammation or pathology)?

Item 2. Cell identity and stemness

- Were cells validated by surface marker expression (e.g., MSC markers)?
- Were negative markers reported (to confirm absence of hematopoietic/endothelial contamination)?
- Was any authentication method used (e.g., STR profiling for human cell lines)?

Item 3. Isolation and culture conditions

- Was the method of isolation specified (enzymatic digestion, explant culture, mechanical separation)?
- Were culture media, supplements, oxygen levels, and serum type reported?
- Were passage number or range provided (to minimize bias from senescence or phenotype drift)?

**Domain 2. Experimental Design**

Item 4. Controls

- Were appropriate negative controls used (e.g., scrambled miRNA, vehicle-only transfection, untreated cells)?
- Were positive controls included when relevant (e.g., known osteogenic inducers such as BMP-2, DEX/β-GP/AA)?
- Were both technical and biological controls applied consistently?

Item 5. Intervention description

- Was the method of miRNA manipulation described in detail (transfection reagent, vector, mimic/inhibitor sequences)?
- Was transfection efficiency validated (e.g., qPCR confirming miRNA overexpression/inhibition)?
- Was the duration of treatment specified and justified?

Item 6. Replicates and reproducibility

- Were biological replicates included (cells from independent donors or independent isolations)?
- Were technical replicates performed (repeated assays within the same sample)?
- Was the number of replicates clearly reported for each experiment?

Item 7. Randomization and blinding (where possible)

- Were samples randomly assigned to treatment groups?
- Were outcome assessors blinded to treatment conditions, or were automated/quantitative methods used to minimize bias?

Item 8. Standardization of conditions

- Were culture conditions standardized across groups (same passage, density, media, serum, incubation time)?
- Were confounding factors (e.g., hypoxia, inflammation, co-culture with bacteria or cytokines) controlled or justified?

**Domain 3. Outcome Assessment**

Item 9. Choice of outcomes

- Were outcomes relevant to dental stem cell biology/regeneration (e.g., proliferation, migration, differentiation into osteogenic/odontogenic lineages)?
- Were both early markers (ALP, RUNX2, DSPP, DMP1) and late markers (mineralization, ARS staining, OCN, COL1) included for differentiation studies?
- Were validated and standardized assays used (e.g., CCK-8/MTT for proliferation, scratch/wound-healing for migration, qPCR/Western blot/ELISA for gene/protein expression)?
- If qualitative assessments were included (e.g., staining images), were they supported by quantitative analysis?

Item 10. Objectivity of assessment

- Was blinding of outcome assessment performed to minimize subjective bias?

Item 11. Normalization

- Were reference genes/proteins for normalization appropriate and justified (e.g., GAPDH, β-actin, U6 for miRNAs)?

**Domain 4. Data Reporting and Analysis**

Item 12. Statistical analysis

- Were the statistical methods described in sufficient detail?
- Was the chosen test appropriate for the type of data (parametric vs non-parametric, multiple comparisons, correction for repeated measures)?
- Was the sample size calculation or justification for replicates provided?

Item 13. Data presentation

- Were numerical results presented with measures of variability (e.g., mean ± SD/SEM, confidence intervals)?
- Were all replicate numbers (n) clearly reported for each experiment?
- Were raw data or representative images (e.g., Western blots, ARS staining, immunofluorescence) shown alongside quantified results?

Item 14. Transparency and selective reporting

- Were results of the negative and positive controls reported in the analysis?
- Were data excluded, and if so, was the reason stated?
- Were all pre-specified outcomes reported, or did the paper selectively highlight favorable results?
- Was there evidence of outcome switching (e.g., reporting some differentiation markers but omitting others without justification)?

**Domain 5. Risk of Bias and Reproducibility**

Item 15. Conflicts of interest and funding transparency

- Was there any potential conflict of interest disclosed (e.g., funding by a company developing miRNA reagents or biomaterials)?
- Was the role of the sponsor in study design, analysis, or reporting described (if applicable)?
- Were funding sources reported?

Item 16. Data availability and transparency

- Were raw data, supplementary datasets, or detailed protocols made available?
- Was data availability sufficient to permit replication by other researchers?

Item 17. Bias acknowledgment

- Did the authors discuss limitations of their study (e.g., single-donor source, lack of in vivo validation, short culture duration)?
- Were potential sources of bias (e.g., batch effects, off-target effects of miRNA mimics/inhibitors) considered?
